# Perceived stress level among patients with chronic illness during covid pandemia

**DOI:** 10.1192/j.eurpsy.2021.768

**Published:** 2021-08-13

**Authors:** M. Pawłowski, K. Fila-Witecka, M. Łuc, A. Senczyszyn, J. Rymaszewska, E. Pawłowska, D. Kamińska, P. Poznański, M. Krajewska, A. Stefaniak, J. Szepietowski, A. Pokryszko-Dragan, S. Budrewicz, T. Pawłowski, J. Rymaszewska

**Affiliations:** 1 Department Of Psychiatry, Wroclaw Medical University, Wroclaw, Poland, Wrocław, Poland; 2 (2)nephrology And Transplantology Department, Wroclaw Medical University, Wroclaw, Poland; 3 Nephrology, Wroclaw Medical University, Wroclaw, Poland; 4 Dermatology, Wroclaw Medical University, Wroclaw, Poland; 5 Neurology Departament, Wroclaw Medical University, Wroclaw, Poland; 6 Department Of Psychiatry, Wroclaw Medical University, Wroclaw, Poland

**Keywords:** Covid, stress, chronic illness

## Abstract

**Introduction:**

The emergence of SARS-CoV-2 has enormously impacted healthcare systems around the world. Both patients and health care professionals have been subjected to a novel stressor which affects their everyday life and functioning. This issue is especially important to patients suffering from chronic diseases which had already been exposed to a psychological strain related to their primary diagnosis. As chronically ill patients are depending on the availability of a specific treatment i.e. in need of specific healthcare facilities and have more reasons to worry about their future and hence be more prone to suffer adverse psychological consequences than the general population.

**Objectives:**

In this study we aimed to examine whether the psychological results of the pandemic affect chronically ill and whether the specific illness and other demographic factors account for any changes in perceived stress levels.

**Methods:**

An online questionnaire has been distributed to 4 groups (n=369): 92 psoriasis patients, 73 dialysis patients, 100 patients after kidney transplantation and 104 multiple sclerosis patients. The study was conducted during the pandemic in Poland (June-July 2020). Perceived stress levels were measured by the Perceived Stress Scale (PSS).

**Results:**

The preliminary results suggest elevated perceived stress levels among the studied groups. As the data are currently under statistical evaluation specific statistical conclusions are to be expected in November 2020.

**Conclusions:**

As the described study was conducted during the SARS-CoV-2 pandemic in Poland, it stands to reason that the epidemiological situation affected the levels of perceived stress among chronically ill patients.

